# β,β-Dimethylacrylalkannin Restores Colistin Efficacy Against *mcr*- and TCS-Mediated Resistant Gram-Negative Bacteria via Membrane Disturbance

**DOI:** 10.3390/antibiotics15010003

**Published:** 2025-12-19

**Authors:** Yongqing Liu, Huangwei Song, Muchen Zhang, Junyao Jiang, Yan Zhang, Jian Xu, Xi Xia, Shangshang Qin, Jianzhong Shen, Yang Wang, Dejun Liu

**Affiliations:** 1 National Key Laboratory of Veterinary Public Health and Safety, College of Veterinary Medicine, China Agricultural University, Beijing 100193, China; liuyongqing@cau.edu.cn (Y.L.); jjy@cau.edu.cn (J.J.); b20233050453@cau.edu.cn (Y.Z.); b20233050493@cau.edu.cn (J.X.); xxia@cau.edu.cn (X.X.); sjz@cau.edu.cn (J.S.); 2 School of Pharmaceutical Science and Technology, Hangzhou Institute for Advanced Study, University of Chinese Academy of Sciences, Hangzhou 310024, China; hwei_song@126.com; 3 Key Laboratory of Advanced Pharmaceutical Technology, Institute of Drug Discovery and Development, School of Pharmaceutical Sciences, Zhengzhou University, Zhengzhou 450001, China; chen_zhang@zzu.edu.cn (M.Z.); qinss@zzu.edu.cn (S.Q.)

**Keywords:** β,β-dimethylacrylalkannin, colistin, membrane dysfunction, LPS transport, efflux pump

## Abstract

**Background:** The reintroduction of colistin has led to the rapid emergence of colistin-resistant strains, significantly diminishing its therapeutic efficacy. This presents a need for effective adjuvants to restore colistin efficacy. **Approach**: We screened the colistin adjuvants through a high-throughput method and then evaluated their synergistic effects and underlying mechanisms. **Results**: We identified β,β-dimethylacrylalkannin (β,β-Dim), a naphthoquinone compound derived from Lithospermum erythrorhizon, as a potent colistin adjuvant (fractional inhibitory concentration index (FICI) < 0.5). β,β-Dim enhanced colistin activity against 4 of 6 susceptible strains and all 18 colistin-resistant strains carrying either plasmid-borne *mcr* genes (*mcr-1*, *mcr-3*, *mcr-8*, and *mcr-9*) or chromosomal two-component system (TCS) mutations (*pmrA*/*B*, *phoP*, and *mgrB*). These strains included *Klebsiella pneumoniae*, *Escherichia coli*, *Salmonella* Typhimurium, *Pseudomonas aeruginosa*, and *Acinetobacter baumannii*. The combination reduced the minimum inhibitory concentrations (MICs) of colistin by 4–1024-fold (from 512 to ≤2 µg/mL). Mechanistically, colistin-mediated outer membrane permeabilization facilitates β,β-Dim entry. Once internalized, β,β-Dim interacts with cytoplasmic membrane phospholipids and disrupts membrane biofunction. Further analysis showed that LPS transport and efflux pump activity were impaired, leading to LPS accumulation in the cytoplasmic membrane and increased intracellular colistin content. These processes elevated reactive oxygen species (ROS) production and markedly reduced ATP levels. In a murine infection model, β,β-Dim (2 mg/kg) combined with colistin (0.2 mg/kg) markedly increased survival from 20% (colistin alone) to 80%. **Conclusions:** These findings highlight that β,β-Dim combined with colistin is a promising therapeutic strategy for infections caused by colistin-resistant pathogens.

## 1. Introduction

Due to the high prevalence of extensively drug-resistant (XDR) Gram-negative bacteria, polymyxins (like colistin) have been reintroduced into clinical practice. However, its reintroduction has led to the rapid emergence of colistin-resistant strains. Resistance develops through the modification of LPS with phosphoethanolamine (PEtN) or 4-amino-4-deoxy-_L_-arabinose (_L_-Ara4N) [1]. These modifications are primarily mediated by TCS mutations, *mgrB* negative feedback, or plasmid-mediated *mcr* gene transfer [2]. These alterations reduce the negative charge of the outer membrane, thereby impairing colistin binding and diminishing its therapeutic efficacy. In response to this challenge, combination therapy has emerged as an effective strategy to enhance antibiotic potency and delay resistance development [3].

Both antimicrobials and non-antibiotic compounds have demonstrated synergistic effects when combined with polymyxins. Antimicrobials include novobiocin [4], tetracyclines [5], etc. Non-antibiotic compounds include *mcr-1* plasmid conjugation inhibitor chelerythrine [6], MCR-1 inhibitor dihydroartemisinin [7], TCS sensor kinase inhibitor IMD-0354 [8], efflux pump inhibitor carbonyl cyanide 3-chloro phenyl hydrazone (CCCP) [9], oxidative stress regulator phenylacylamide derivative A22 [10], etc. These adjuvants either possess intrinsic activity against Gram-negative bacteria or disrupt essential bacterial physiological processes. While these combinations can enhance the antibacterial efficacy of polymyxins, the use of antibiotics carries the risk of cross-resistance [11]. These inhibitors exhibit narrow target specificity, limited activity against non*-mcr-1* variants or chromosomal-mediated resistant bacteria, off-target effects on TCS, and often high cytotoxicity. TCS mutations represent the most common mechanism of colistin-resistant bacteria, particularly *K. pneumoniae* [12]. Notably, antimicrobials selective for Gram-positive bacteria, such as linezolid and α-mangostin, have also shown synergy with polymyxins for Gram-negative bacteria [13,14]. Leveraging anti-Gram-positive agents as colistin adjuvants may complement colistin’s mechanism of action and provide broad-spectrum synergistic antibacterial activity.

Natural compounds, known for their structural diversity and broad biological activity, are a major source of novel drug candidates [15]. Considering that anti-Gram-positive agents can exhibit synergy with colistin against Gram-negative bacteria, the study aimed to identify natural compounds with anti-Gram-positive bacteria activity, low toxicity, and efficient synergistic effects when combined with colistin. β,β-dimethylacrylalkannin (β,β-Dim), a naphthoquinone compound, was identified as a colistin potentiator. However, the synergistic effect and mechanism of β,β-Dim with colistin remain unclear. In this study, we evaluated the synergistic activity of the β,β-Dim–colistin combination and analyzed its synergistic mechanism.

## 2. Results

### 2.1. β,β-Dim Is Identified as a Potential Colistin Adjuvant Through High-Throughput Screening

**Figure 1:**
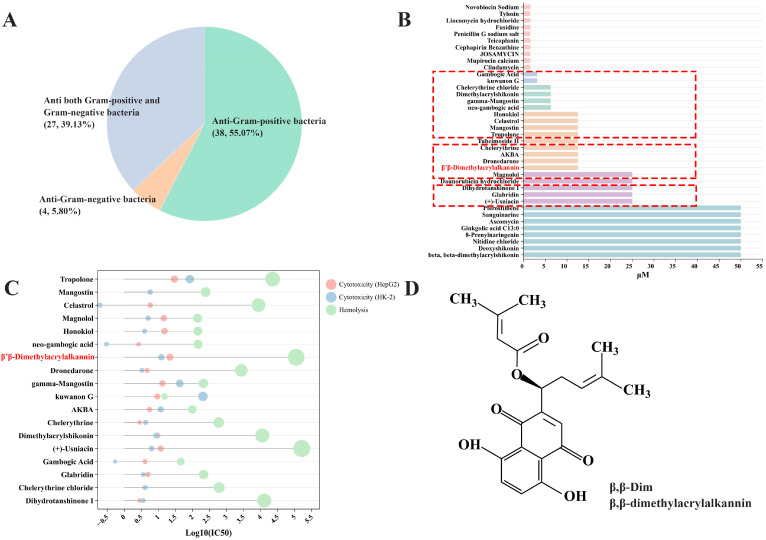
High-throughput screening of β,β-Dim. (**A**) Identification of 38 anti-Gram-positive bacteria compounds from a natural compound library. (**B**) MICs of the 38 compounds. A total of 18 compounds (highlighted in red frame) showed MICs < 10 μg/mL. (**C**) Cytotoxicity and hemolytic activity of the 18 compounds. (**D**) Chemical structure and diagram of β,β-Dim.

To identify efficient and low-toxicity anti-Gram-positive compounds as colistin adjuvants, a systematic screening was conducted using a natural compound library. Firstly, 38 compounds with antibacterial activity against *S. aureus* ATCC 29213 were selected from a library of 2462 compounds (50 µM) (Figure 1A). Further, MIC values of the 38 compounds were determined. Excluding 10 antibiotics, 18 compounds with MICs < 10 μg/mL (highlighted in red) were selected (Figure 1B). Cytotoxicity and hemolytic activity were assessed, with most compounds showing log_10_(IC_50_) ranging from 0.5 to 5.5 (Figure 1C). Based on criteria of IC_50_ > 10 µM for both hemolysis and cytotoxicity, and excluding compounds with prior reports or known toxic features, β,β-Dim was ultimately identified as a promising colistin adjuvant (Figure 1D).

**Figure 2:**
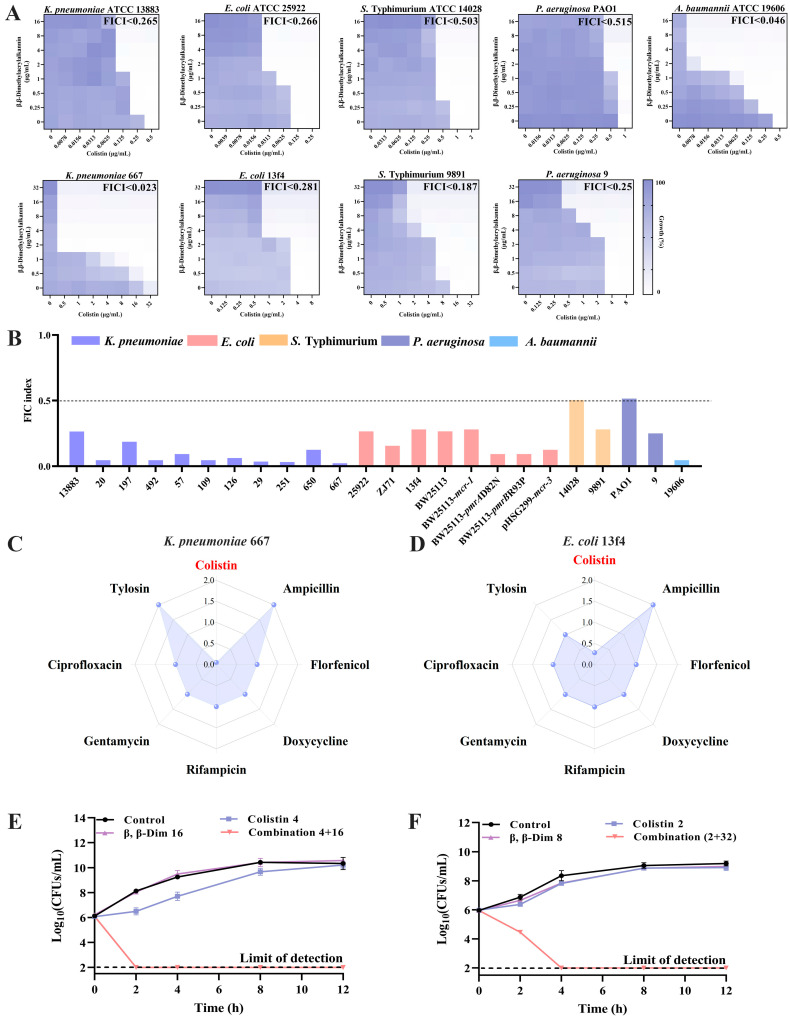
Synergistic antibacterial activity of β,β-Dim combined with colistin. (**A**) Checkerboard assays evaluating the synergy. (**B**) FICIs for all tested strains. (**C**,**D**) The FICIs of β,β-Dim combined with different antibiotics against *K. pneumoniae* 667 and *E. coli* 13f4. (**E**,**F**) Time-kill kinetics of the combination against *K. pneumoniae* 667 and *E. coli* 13f4. COL: colistin. Data represent three biological replicates.

To assess the synergistic effect of colistin and β,β-Dim, 24 bacterial strains were selected, including *K. pneumoniae*, *E. coli*, *Salmonella*, *P. aeruginosa*, and *A. baumannii*. There were 6 susceptible strains, 13 *mcr*-positive strains (*mcr*-*1*, *mcr*-*3*, *mcr*-*8*, and *mcr*-*9*), and 5 strains harboring mutations in either the TCS (*pmrA*/*B*, *phoP*) or *mgrB*. Among the six susceptible strains, β,β-Dim combined with colistin exhibited synergistic effects against *K. pneumoniae* (FICI < 0.265), *E. coli* (FICI < 0.266), and *A. baumannii* (FICI < 0.046) without synergistic effects against *Salmonella* ATCC 14028 or *P. aeruginosa* PAO1 (FICI > 0.5). The MICs of colistin were reduced by 4–64-fold. In the 18 colistin-resistant strains (MICs ranging from 4 to 512 μg/mL), the combination also demonstrated synergistic activity. In *K. pneumoniae* (including *mcr-1*, *mcr-8*, *mcr-9*, *pmrA*/*B*, *phoP*, and *mgrB*), 70% (n = 7/10) were also resistant to carbapenems (*bla*_NDM_ or *bla*_KPC_), and colistin activity was enhanced by 8–1024-fold (FICI < 0.187). In *E. coli* (including *mcr-1*, *mcr-3*, and *pmrA*/*B*), colistin efficacy improved by 4–32-fold (FICI < 0.281). In *Salmonella* and *P. aeruginosa* (*mcr-1*), enhancement ranged to 8-fold (FICI < 0.187 and <0.25, respectively) (Figure 2A,B, Table S2). Overall, the β,β-Dim–colistin combination demonstrates broad-spectrum efficacy against diverse resistant bacteria, consistently lowering the colistin MIC to ≤ 2 μg/mL.

To assess whether β,β-Dim possesses broad-spectrum synergistic effects, FICIs were determined for its combination with seven antibiotics against *K. pneumoniae* 667 (*phoP*, *pmrA*) and *E. coli* 13f4 (*mcr-1*). Checkerboard assay revealed synergy exclusively with colistin (*K. pneumoniae*: FICI < 0.023, *E. coli*: FICI < 0.281), while no synergistic interactions were observed with ampicillin, florfenicol, doxycycline, rifampicin, gentamicin, ciprofloxacin, or tylosin (FICI > 1) (Figure 2C,D). To verify the synergistic effect of this combination, bacterial growth and time-killing assays were performed. Growth curves showed that neither β,β-Dim (4 µg/mL) nor colistin (0.5 µg/mL) alone inhibited the growth of *K. pneumoniae* 667, whereas their combination completely suppressed bacterial proliferation. Similar effects were observed in *E. coli* 13f4 (Figure S1). Time-kill assays further confirmed the bactericidal synergy: the combination reduced bacterial counts by 4 log_10_ CFUs/mL for *K. pneumoniae* 667 at 2 h and 5.5 log_10_ CFUs/mL for *E. coli* 13f4 at 4 h, compared to colistin alone (Figure 2E,F). Together, these results support β,β-Dim as a promising colistin adjuvant capable of restoring colistin efficacy against resistant Gram-negative bacteria.

### 2.2. β,β-Dim Enhances Colistin Efficacy by Inhibiting LPS Transport and Efflux Pump Activity, and by Increasing Intracellular Colistin Accumulation

**Figure 3:**
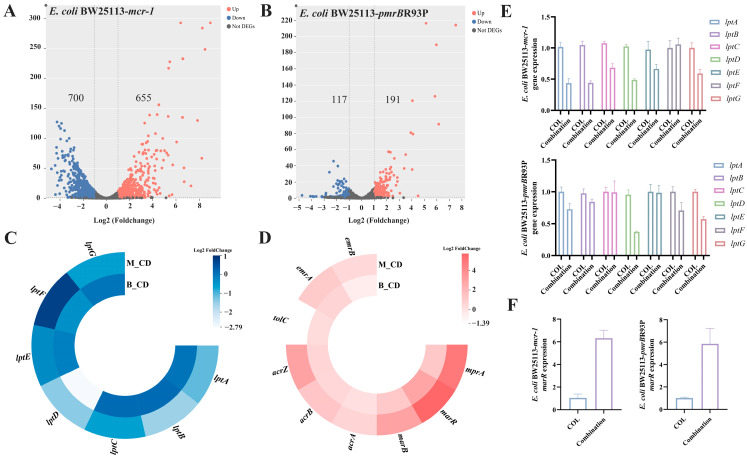
Transcriptomic analysis of the synergistic mechanism between β,β-Dim and colistin. (**A**) Volcano plot of DEGs in *E. coli* BW25113-*mcr-1* treated with β,β-Dim (8 μg/mL) and colistin (2 μg/mL) for 1 h. (**B**) Volcano plot of DEGs in *E. coli* BW25113-*pmrB*R93P treated with β,β-Dim (8 μg/mL) and colistin (4 μg/mL) for 1 h. Key metabolic pathways associated with common DEGs following the combination treatment include the following: (**C**) LPS transport and (**D**) MarR family. M_CD and B_CD represented DEGs in the combination group compared to colistin alone in *E. coli* BW25113-*mcr-1* and *E. coli* BW25113-*pmrB*R93P, respectively. (**E**,**F**) RT-qPCRvalidation. COL: colistin. Data represent three biological replicates.

To elucidate the mechanism underlying the synergistic effect of β,β-Dim and colistin, transcriptomic analyses were performed on *E. coli* BW25113-*mcr-1* and *E. coli* BW25113-*pmrB*R93P following treatment with 8 μg/mL β,β-Dim in combination with 2 or 4 μg/mL colistin. Based on the criteria of *q* < 0.05 and |log2 (fold change)| > 1, 1355 differentially expressed genes (DEGs) were identified in *E. coli* BW25113-*mcr-1* and 308 DEGs in *E. coli* BW25113-*pmrB*R93P (Figure 3A,B). Common DEGs between the two strains were enriched in genes related to LPS transport and the multiple antibiotic resistance regulator (MarR) family (Figure 3C,D). RT-qPCR validation confirmed significant downregulation of *lptD* and upregulation of *marR* expression in the combination group compared with colistin alone (Figure 3E,F).

**Figure 4:**
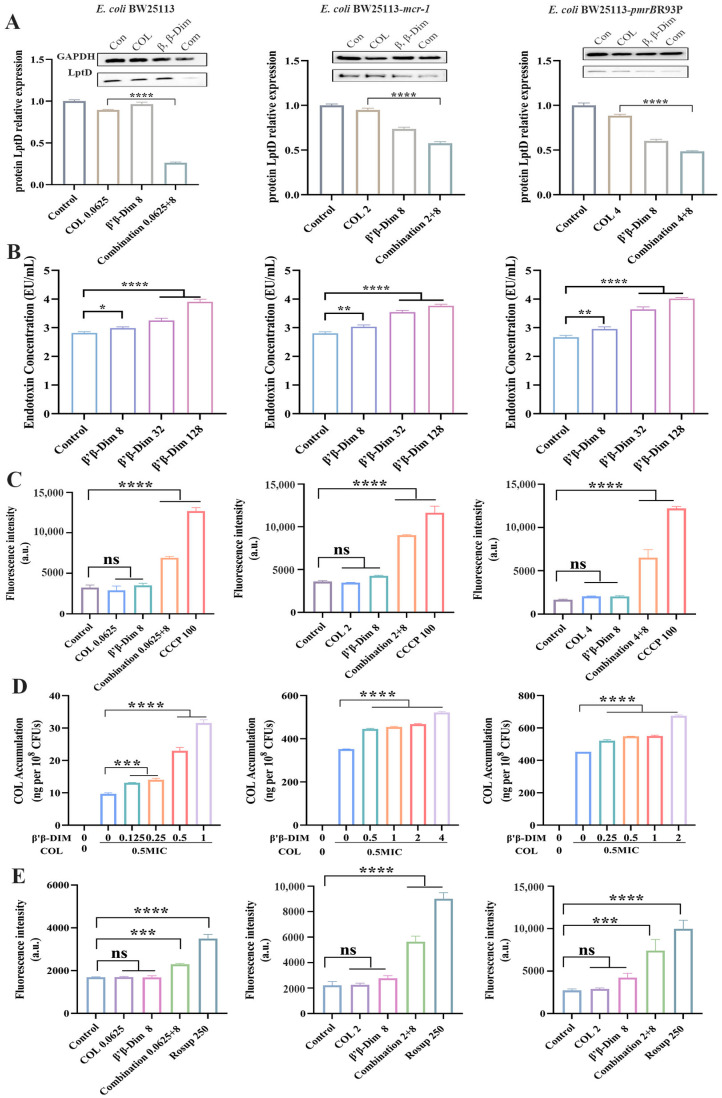
β, β-Dim enhances the antibacterial activity of colistin. (**A**) LptD protein expression. (**B**) LPS concentrations in the cytoplasmic membrane. (**C**) Efflux pump activity, assessed by intracellular EB fluorescence intensity. CCCP was used as a positive control. (**D**) Intracellular colistin levels after exposure to varying concentrations of β,β-Dim. (**E**) ROS levels. Rosup served as a positive control. COL: colistin. Unit: μg/mL. COL: colistin. Data represent three biological replicates. ns, no significant difference. * *p* < 0.05, ** *p* < 0.01, *** *p* < 0.001, **** *p* < 0.0001.

As the terminal component of the LPS transport system, LptD receives LPS from the periplasmic protein LptA and, in association with LptE, anchors it to the outer leaflet of the outer membrane [16]. Downregulation of *lptD* is expected to hinder LPS translocation to the outer membrane, thereby affecting colistin–bacteria interactions. Western blot analysis demonstrated that β,β-Dim combined with colistin significantly reduced LptD expression to approximately two-fold lower than that in the colistin group in *E. coli* BW25113, *E. coli* BW25113-*mcr-1*, and BW25113-*pmrB*R93P (Figure 4A). Consistent with a previous report showing that the LptD inhibitor Murepavadin induces LPS accumulation in the cytoplasmic membrane and enhances colistin susceptibility [17], β,β-Dim–colistin treatment increased LPS levels in the cytoplasmic membrane in a concentration-dependent manner (Figure 4B).

MarR acts as a transcriptional repressor, binding to the *marO* operator to inhibit the expression of *marA*, thereby limiting AcrAB-TolC efflux pump activity [18]. The combination of colistin with the efflux pump inhibitor CCCP has been reported to potentiate colistin activity, reducing its MIC by approximately 8-fold [19]. To assess efflux pump function, the DNA-intercalating fluorescent dye EB was utilized. Under normal efflux activity, EB is efficiently expelled, resulting in minimal intracellular fluorescence. In contrast, EB fluorescence intensity was markedly increased in the β,β-Dim–colistin combination group, indicating impaired efflux pump function (Figure 4C). Given the observed increase in LPS content in the cytoplasmic membrane and the inhibition of efflux pump activity, we hypothesized that intracellular colistin accumulation would be elevated. As expected, β,β-Dim enhanced intracellular colistin levels in a dose-dependent manner, exceeding 0.6 µg per 10^8^ CFUs at the highest tested concentration (Figure 4D). It has been demonstrated that colistin induces bacterial cell death through the ROS pathway [2]. The combination of β,β-Dim with colistin markedly elevated intracellular ROS levels by approximately 1.35-fold, 3.62-fold, and 3.53-fold, respectively, further supporting their synergistic antimicrobial activity (Figure 4E).

These findings suggest that β,β-Dim enhances colistin activity by disrupting LPS transport and promoting the accumulation of LPS in the cytoplasmic membrane. Concurrently, the combination impairs efflux pump activity, thereby increasing intracellular colistin accumulation and inducing ROS overproduction, ultimately leading to bacterial cell death.

### 2.3. β,β-Dim Disrupts Biofunction of Cytoplasmic Membrane by Targeting Phospholipids

Gene Ontology (GO) enrichment analysis revealed that, compared to colistin monotherapy, DEGs in the combination group were primarily associated with ATP metabolism, NADH dehydrogenase activity, and oxidoreductase activity (Figure S2A–D). Kyoto Encyclopedia of Genes and Genomes (KEGG) analysis showed enrichment in oxidative phosphorylation and ABC transporter pathways (Figure S3A–D), suggesting the combination disrupts the biofunction of cytoplasmic membrane and bacterial energy metabolism.

**Figure 5:**
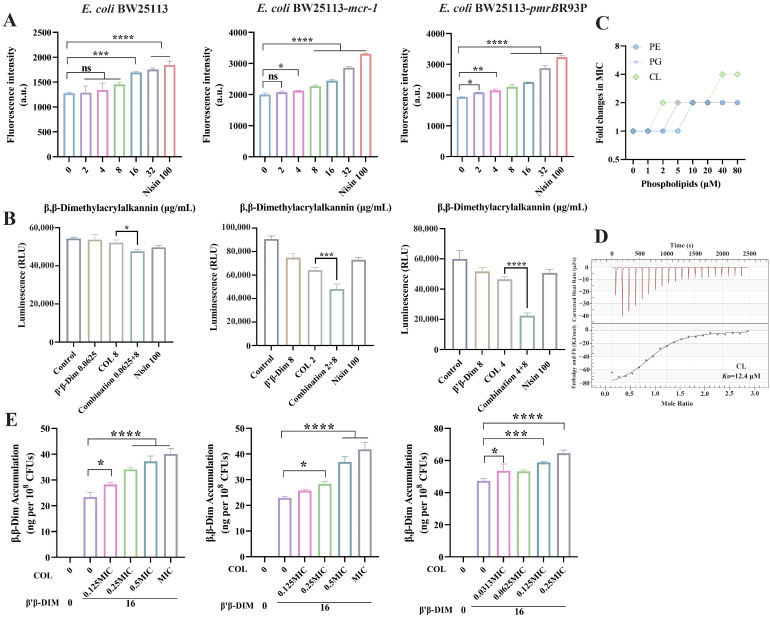
β,β-Dim targets the phospholipids and disrupts cytoplasmic membrane biofunction. (**A**) Effects of β, β-Dim on membrane permeability in *E. coli* spheroplasts. (**B**) Intracellular ATP level in *E. coli*. (**C**) Influence of PE, PG, and CL on the antibacterial activity of β, β-Dim against *S. aureus* ATCC 29213. (**D**) Binding affinity of β, β-Dim for CL measured by ITC assays. (**E**) Intracellular β,β-Dim levels in *E. coli* BW25113, *E. coli* BW25113-*mcr-1*, and *E. coli* BW25113-*pmrB*R93P following treatment with varying concentrations of colistin. Data represent three biological replicates. COL: colistin. Unit: μg/mL. ns, no significant difference. * *p* < 0.05, ** *p* < 0.01, *** *p* < 0.001, **** *p* < 0.0001.

Given the potent activity of β,β-Dim against Gram-positive bacteria, the effect of β,β-Dim on membrane permeability was first evaluated in *S. aureus* ATCC 29213. β,β-Dim increased membrane permeability in a concentration-dependent manner, as indicated by elevated PI fluorescence intensity (Figure S4A). Thus, we speculated that β,β-Dim may similarly affect the cytoplasmic membrane of Gram-negative bacteria. To investigate this, *E. coli* spheroplasts were prepared to remove the outer membrane barrier. Cells stained with SYTO9 displayed typical rod-shaped morphology, whereas spheroplasts appeared spherical under CLSM, confirming successful preparation (Figure S4B). Consistently, β,β-Dim markedly increased membrane permeability in *E. coli* spheroplasts (Figure 5A), suggesting a direct interaction with the membrane. Meanwhile, β,β-Dim increased membrane potential in *S. aureus* ATCC 29213, reduced the proton gradient, and dissipated the PMF (Figure S4C). Since transmembrane proton transport generates the PMF that drives ATP synthesis, disruption of this process led to a marked decrease in intracellular ATP levels by approximately 1.09-fold, 1.33-fold, and 2.07-fold, respectively, in *E. coli* following combined treatment with β,β-Dim and colistin (Figure 5B). To investigate this interaction, the effects of membrane components on the antibacterial activity of β,β-Dim were examined. Exogenous addition of PE, PG, and CL attenuated the antibacterial activity of β,β-Dim against *S. aureus* ATCC 29213, with CL (40 μM) increasing the MIC by 4-fold (Figure 5C). Similarly, phospholipids diminished the synergistic effect of β,β-Dim and colistin in *E. coli* (Figure S4D). The interaction was further validated through ITC assays, which demonstrated that β,β-Dim exhibited the strongest affinity for CL (*K*_D_ = 12.4 μM), with an exothermic and spontaneous binding process (Δ*H* < 0, Δ*G* < 0) (Figure 5D and Figure S4E). As β,β-Dim alone did not exhibit antibacterial activity against Gram-negative bacteria, and considering that colistin disrupts the outer membrane, we hypothesized that this disruption facilitated β,β-Dim entry into the cells, enabling it to interact with the cytoplasmic membrane. To verify this, intracellular β,β-Dim levels were quantified by HPLC-MS/MS in *E. coli* BW25113, *E. coli* BW25113-*mcr-1*, and *E. coli* BW25113-*pmrB*R93P. Co-treatment with colistin significantly enhanced β,β-Dim intracellular accumulation across all three strains in a concentration-dependent manner, reaching approximately 60 ng per 10^8^ CFUs (Figure 5E). These findings suggest that colistin-mediated permeabilization of the outer membrane facilitates β,β-Dim entry into the cells. Once internalized, β,β-Dim interacts with phospholipids, disrupts the cytoplasmic membrane biofunction, and subsequently inhibits LPS transport and efflux pump activity, thus exerting a synergistic antibacterial effect with colistin.

### 2.4. β,β-Dim Enhances Colistin’s Therapeutic Efficiency In Vivo

**Figure 6:**
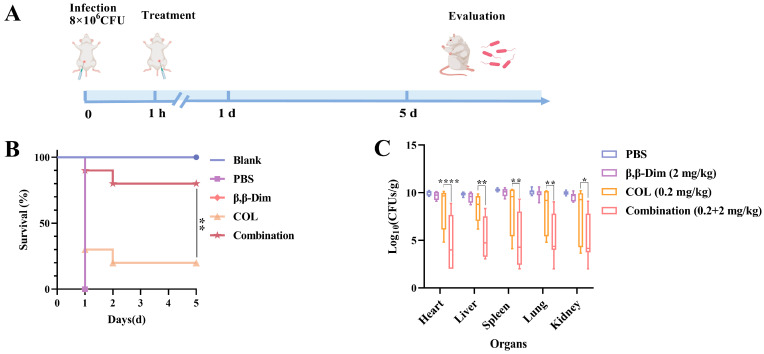
Therapeutic efficacy of β,β-Dim combined with colistin in a murine systemic infection model induced by intraperitoneal injection of *K. pneumoniae* 667. (**A**) Schematic diagram of the experimental protocol. (**B**) Survival rates of each treatment group (n = 10) following a single therapeutic intervention. (**C**) Bacterial burdens in major organs of each treatment group (n = 10). COL: colistin. * *p* < 0.05, ** *p* < 0.01, **** *p* < 0.0001.

Based on the effective synergistic antibacterial effect in vitro, a murine systemic infection model was established to evaluate the in vivo therapeutic efficacy of the β,β-Dim–colistin combination (Figure 6A). *K. pneumoniae*, an opportunistic pathogen that colonizes the respiratory tract, gastrointestinal tract, urinary tract, and bloodstream, was selected due to its clinical relevance and global public health importance [20]. As shown in Figure 6B, combination therapy improved the survival rate, increasing the survival rate from 20% with colistin monotherapy to 80% at day 5 post-infection. In parallel, bacterial loads in the heart, liver, spleen, lung and kidneys were significantly reduced by 1.5–6 Log10 CFUs/g following treatment with β,β-Dim (2 mg/kg) in combination with colistin (0.2 mg/kg) (Figure 6C). Histopathological analysis further supported these findings. Mice in the PBS group exhibited severe tissue damage, including cardiac edema, interstitial widening, hepatic necrosis, inflammatory infiltration, spleen pulp atrophy, alveolar congestion, alveolar wall thickening, and renal interstitial congestion. Although colistin group partially alleviated these lesions, the combination treatment substantially mitigated tissue damage (Figure S5). These results demonstrate that β,β-Dim enhances the therapeutic efficiency of colistin in vivo.

## 3. Discussion

Reports of colistin-resistant pathogenic bacteria have increased rapidly worldwide [21]. The coexistence of colistin resistance with other antibiotic resistance has further compromised the therapeutic efficacy of colistin [22]. Encouragingly, this study identified β,β-Dim, a naphthoquinone derivative with anti-Gram-positive activity, as a potent colistin adjuvant. The β,β-Dim–colistin combination displayed robust synergistic antibacterial activity against a broad range of colistin-resistant Gram-negative bacteria, including both plasmid-borne (*mcr-1*, *mcr-3*, *mcr-8*, and *mcr-9*) and chromosome-encoded (*pmrA*/*B*, *phoP*, and *mgrB*) resistant strains of *E. coli*, *K. pneumoniae*, *Salmonella*, and *P. aeruginosa*. In contrast to other colistin adjuvants, such as anti-alcoholism drug disulfiram and cajanin stilbene acid, they primarily target *mcr*-positive bacteria [23,24]. This broader activity spectrum suggests that β,β-Dim may offer advantages over MCR inhibitors and potentially extend the clinical utility of colistin. In comparison, the broad-spectrum adjuvant SLAP-S25 failed to exhibit synergistic activity against *mcr*-positive *K. pneumoniae* when combined with colistin [25]. In this study, β,β-Dim markedly potentiated colistin activity against *K. pneumoniae* strains harboring *mcr-1*, *mcr-8*, *mcr-9*, *pmrA*, *phoP*, or *mgrB* mutations, with FICI < 0.187 and reduction in colistin MICs ranging from 8- to 1024-fold. Notably, these strains also carried carbapenem resistance genes *bla*_NDM_ or *bla*_KPC_, representing clinically significant extensively drug-resistant (XDR) pathogens. The emergence of *K. pneumoniae* strains resistant to both colistin and carbapenems has become a major cause of hospital-acquired infections associated with high morbidity and mortality [26]. Remarkably, in a murine systemic infection model established by colistin- and carbapenem-resistant *K. pneumoniae* 667 (*phoP*, *pmrA*, and *bla*_KPC_), the β,β-Dim–colistin combination significantly reduced the bacterial loads in major organs and improved survival, underscoring its potential as a promising therapeutic option for XDR bacterial infections.

β,β-Dim, a naphthoquinone alkannin derivative from *Lithospermum erythrorhizon*, exhibits high lipophilicity and multiple biological activities, including antibacterial, antioxidant, anti-inflammatory, and wound healing properties [27]. Its precise antimicrobial mechanism remains unclear. Naphthoquinone compounds are reported to act against multidrug-resistant Gram-positive bacteria (e.g., methicillin-resistant *S. aureus*, vancomycin-resistant *Enterococcus*, and linezolid-resistant *Enterococcus*) by disrupting membrane integrity, dissipating the PMF, inhibiting the respiratory chain, and reducing ATP synthesis [28]. They can also inhibit ATP synthase, suppressing the growth of *E. coli* strains with wild-type or mutant ATP synthase, and limit resistance plasmid dissemination (IncI2/IncX4 carrying *mcr-1*, IncX4/IncX3 harboring *bla*_NDM-5_, IncFI/IncFII containing *tet*(X4)) [29,30]. Additionally, naphthoquinone compounds inhibit topoisomerase I and xanthine oxidase [31,32]. Here, we demonstrated that β,β-Dim interacts with cardiolipin to disrupt membrane permeability, causing PMF dissipation and energy depletion. In combination with colistin, it blocks LPS transport and efflux pump function, leading to LPS accumulation in the cytoplasmic membrane, restoring colistin susceptibility and enhancing intracellular colistin levels and bactericidal activity. Unlike neomycin, which potentiates polymyxin via LptB binding [4], β,β-Dim reduces LptD synthesis and blocks LPS transport. Although both β,β-Dim and murepavadin inhibit LPS transport and cause LPS accumulation in the cytoplasmic membrane, the latter exerts its effect by directly binding to LptD [17]. Furthermore, the combination of β,β-Dim and colistin decreased bacterial ATP levels, elevated intracellular ROS production, and induced broad-spectrum antimicrobial activity against bacteria with different resistance phenotypes (*mcr*-positive and TCS-mutant strains). These findings further indicate that β,β-Dim may serve as a more effective colistin adjuvant than agents with a single mode of action.

Beyond its antibacterial properties, β,β-Dim also exhibits anti-tumor and immunomodulatory activities [33], with minimal associated toxicity. Previous studies have reported that the median lethal dose (LD_50_) of β,β-Dim exceeds 10 g/kg in acute oral toxicity tests in mice, that its maximum tolerable dose also exceeds 10 g/kg in rats, and that the no-observed-adverse-effect level (NOAEL) of β,β-Dim is 10 mg/kg in rats [34]. These indicate that β,β-Dim possesses favorable safety in mice. However, the toxicity associated with higher or repeated doses, as well as selectivity, still requires further investigation in our study. Additionally, intraperitoneal administration of β,β-Dim (2 mg/kg) combined with colistin (0.2 mg/kg) significantly improved mouse survival to 80%, suggesting favorable safety in vivo. Although β,β-Dim does not exhibit significant toxicity, its metabolism fate remains unclear. Given the disparity between in vivo and in vitro effective concentrations, as well as the compound’s structural features, it is likely that β,β-Dim undergoes metabolic transformation or displays pharmacokinetic differences relative to colistin. These processes may ultimately reduce synergistic potency. Additionally, the colistin dose used (0.2 mg/kg) is substantially lower than the maximum recommended doses (polymyxin B: 1.5–2.5 mg/kg, CMS: 6 MU, colistin: 9 MU) and is expected to minimize nephrotoxicity [35]. Nevertheless, further research is needed to clarify the detailed toxicity profile of β,β-Dim, evaluate the efficacy of the β,β-Dim–colistin combination in diverse infection models, and characterize their pharmacokinetic properties.

## 4. Materials and Methods

### 4.1. Compounds, Antibiotics, and Mice

The natural compounds library (Cat No. L6000) and β,β-Dim (purity > 95%) were purchased from TargetMol Chemicals Inc. (Boston, MA, USA). COL, ampicillin, florfenicol, doxycycline, rifampicin, gentamycin, ciprofloxacin, and tylosin were purchased from Aladdin Biochemical Technology Co., Ltd. (Shanghai, China). Female BALB/c mice weighing 20 ± 2 g and aged 8 weeks were obtained from Beijing Vital River Laboratory Animal Technology Co., Ltd. (Beijing, China).

### 4.2. Cell-Based Screening

A total of 2462 natural compounds were screened by determining the MIC according to the Clinical and Laboratory Standards Institute (CLSI) guideline (M100). Briefly, natural compounds at a final concentration of 50 µM were co-incubated with bacterial suspensions. Following 18 h incubation at 37 °C, MICs were determined by measuring OD600 using an Infinite M200pro microplate reader (Tecan, Männedorf, Switzerland).

### 4.3. Toxicity Assay

Toxicity assessment was performed by using sheep blood cells, human renal proximal tubular epithelial HK-2, and human hepatocarcinoma HepG2. Briefly, 8% erythrocyte suspensions were incubated with the test compounds (0–250 µM) for 1 h at 37 °C. After centrifugation, supernatants were transferred to a 96-well plate, and absorbance was measured at 570 nm.

The HepG2 and HK-2 cells (10^4^ cells/well) were cultured in DMEM medium containing 10% fetal bovine serum (FBS) and then placed in a 5% CO_2_ incubator for 24 h. The culture medium was replaced with fresh DMEM medium containing the compounds (0–250 µM), followed by a further 24 h incubation. Subsequently, 10 µL of CCK-8 was added and incubated for 1 h, and OD450nm values were measured.

Hemolysis% = [OD570nm_(drug)_ − OD570nm_(blank)_]/[OD570nm_(TritonX−100)_ − OD570nm_(blank)_] × 100%

Cell viability% = [OD450nm_(drug)_ − OD450nm_(blank)_]/[OD450nm_(negative)_ − OD450nm_(blank)_] × 100%

### 4.4. Checkerboard Assay

The FICI of β,β-Dim–colistin combination was determined by a checkerboard assay. Briefly, two-fold serial dilutions of β,β-Dim and colistin were prepared in a 96-well plate. After incubation at 37 °C for 18 h with bacterial suspension (10^6^ CFU/mL), OD600 values were measured. FICI = (MIC_β,β-Dim in combination_/MIC_β,β-Dim_) + (MIC_colistin in combination_/MIC_colistin_). The synergistic effect of β,β-Dim with other antibiotics was measured in the same way. Synergy was considered to be an FICI ≤ 0.5.

### 4.5. Antibacterial Activity

Overnight cultures of *K. pneumoniae* 667 and *E. coli* 13f4 were diluted to 10^6^ CFU/mL; then, β,β-Dim (4 μg/mL), colistin (0.5 or 2 μg/mL), and their combination were co-incubated with bacterial suspensions in a 96-well plate. OD600 values were monitored hourly over a 24 h period.

The killing kinetics were measured by enumerating bacterial colonies. Briefly, *K. pneumoniae* 667 and *E. coli* 13f4 (10^6^ CFU/mL) were incubated with β,β-Dim (16 or 32 μg/mL), colistin (2 or 4 μg/mL), and their combination under continuous shaking. Subsequently, the cultures were collected, diluted, and plated on MHA plates at 0, 2, 4, 8, and 12 h. Finally, colony-forming units (CFUs) were counted.

### 4.6. Transcriptome Analysis

*E. coli* BW25113-*mcr-1* and *E. coli* BW25113-*pmrB*R93P bacterial suspensions were washed and suspended with PBS to an OD600 of 0.5. For the *E. coli* BW25113-*mcr-1* group, colistin (2 µg/mL) and colistin combined with β,β-Dim (2 + 8 µg/mL) were added to the bacterial suspension. The *E. coli* BW25113-*pmrB*R93P group was treated similarly, with a final colistin concentration of 4 µg/mL. After co-incubation for 1 h, bacterial pellets were collected, and total RNA was extracted using an RNA extraction kit (Cat No. AG21017, Accurate, Changsha, China), and RNA concentrations were determined using a NanoDrop 2000 (Thermo Fisher, Waltham, MA, USA). cNDA Library construction, high-throughput sequencing, data analysis, and functional enrichment analysis were conducted by Sinobiocore Biotechnology Co., Ltd. (Beijing, China). The analysis results were verified through reverse transcription–quantitative polymerase chain reaction (RT-qPCR) by using QuantStudio^TM^ 7 Flex (Applied Biosystems, Foster City, CA, USA). Relative gene expression was calculated using 2^−ΔΔCT^, with 16S rRNA as the internal reference. The primer sequences are listed in Table S1.

### 4.7. LptD Expression

Bacterial cultures (OD600 = 0.5) of *E. coli* BW25113, *E. coli* BW25113-*mcr-1*, and *E. coli* BW25113-*pmrB*R93P carrying a FLAG tag were treated with β,β-Dim, colistin, or their combination for 1 h. After incubation, the bacterial total proteins were extracted using a complete bacterial protein extraction reagent (Cat No. 89822, Thermo Fisher, Waltham, MA, USA). Protein concentrations were measured with the BCA Protein Assay Kit (Cat No. 23227, Thermo Fisher, Waltham, MA, USA). Subsequently, protein samples were boiled together with 4× protein loading buffer, and equal amounts of protein (20 µg) were separated by SDS-PAGE (12.5%) and transferred to a PVDF membrane. Membranes were blocked for 2 h, washed, incubated with antibodies (anti-GAPDH, Cat No. ab9484, Abcam, Cambridge, UK; anti-FLAG antibody, Cat No. F3165, Sigma-Aldrich, Saint Louis, MO, USA) overnight at 4 °C, washed again, and then incubated with an Anti-Mouse IgG (H+L)–peroxidase antibody produced in rabbit (Cat No. SAB3701083, Sigma-Aldrich, Saint Louis, MO, USA) for 1 h. Following washes, signals were developed with ECL Master Mix (Cat No. E412, Vazyme, Nanjing, China), and protein bands were visualized using a gel imaging system (Tanon-5200, Bio-Tanon, London, UK).

### 4.8. Determination of LPS Concentrations

The cytoplasmic membrane LPS concentrations of *E. coli* BW25113, *E. coli* BW25113-*mcr-1*, and *E. coli* BW25113-*pmrB*R93P were quantified using a Chromogenic LAL Endotoxin Assay Kit (Cat No, C0273S, Beyotime, Shanghai, China). Firstly, spheroplasts were prepared according to a previously described method to remove the outer membrane [17]. Briefly, bacterial cells were washed with Tris buffer and resuspended in Tris buffer containing 20% sucrose. EDTA (250 μL, 10 mg/mL) and lysozyme (1 mL, 10 mg/mL) were added, followed by incubation at 30 °C for 1 h with shaking at 80 rpm. Trypsin (500 μL, 10 mg/mL) was added and incubated for an additional 15 min. Spheroplasts were harvested by centrifugation at 4000 rpm for 20 min at 4 °C, and their formation was confirmed by morphological examination using a confocal laser scanning microscope (CLSM) SP8 (Leica, Wetzlar, Germany). In this study, spheroplasts were prepared by incubating β,β-Dim (0, 8, 32, 128 µg/mL), followed by repeated freeze–thaw to release LPS. Then, the released LPS was quantified by reacting with LPS detection reagents, and absorbance was measured at 405 nm.

### 4.9. Efflux Pump Activity

Efflux pump activity in *E. coli* treated with β,β-Dim and colistin was evaluated using ethidium bromide (EB, Cat No. E808961, Macklin, Shanghai, China). Bacterial cultures (OD600 = 0.5) were incubated with β,β-Dim, colistin, or their combination for 1 h. After incubation, cells were collected by centrifugation, washed with PBS, and resuspended in PBS containing 4 µg/mL EB for 5 min. Fluorescence was measured at excitation 530 nm and emission at 600 nm.

### 4.10. Intracellular Compound Content Determination

Overnight cultures *E. coli* BW25113, *E. coli* BW25113-*mcr-1*, and *E. coli* 1 BW25113-*pmrB*R93P were diluted to 10^8^ CFU/mL and then co-incubated with colistin (0.5 × MIC) in the presence of β,β-Dim (0–4 μg/mL) for 30 min at 37 °C. The cultures were centrifuged and resuspended in 30% methanol. Bacteria were disrupted by sonication and centrifugation, supernatants were diluted in 20% acetonitrile, and intracellular colistin was quantified via HPLC-MS/MS (TQ-XS, Waters, Milford, MA, USA). Analyses were performed on a CORTECS Shield RP C18 column (Waters, Milford, MA, USA) at 40 °C, with a flow rate of 0.4 mL/min and an injection volume of 5 μL. The mobile phases consisted of solvent A (0.5% formic acid and acetonitrile in water) and solvent B (0.5% formic acid in water). Colistin was detected in positive ESI mode with ion transitions *m*/*z* 368.0 > 380.1 and *m*/*z* 386.0 > 101.0. Intracellular β,β-Dim content was determined using the same procedure, while bacterial suspensions were co-incubated with β,β-Dim (16 μg/mL) and colistin at 0–1 × MIC. Mobile phases included solvent A (acetonitrile) and solvent B (water). β,β-Dim was detected in the negative electrospray ionization (ESI) mode with ion transitions *m*/*z* 369.1 > 269.0 and *m*/*z* 369.1 > 241.0.

### 4.11. ROS Determination

The levels of ROS in *E. coli* treated with β,β-Dim–colistin combination were measured with 2′,7′-dichlorofluorescein diacetate (DCFH-DA, Cat No. S1105S, Beyotime, Shanghai, China). Bacterial suspensions (OD600 = 0.5) were incubated with 10 μM DCFH-DA for 30 min, and then centrifuged and resuspended in PBS. β,β-Dim, colistin, or their combination groups were incubated with fluorescently labeled bacterial suspension for 1 h, and fluorescence was measured at an excitation/emission of 488/525 nm.

### 4.12. Membrane Permeability Determination

The *S. aureus* ATCC 29213 bacterial suspension (OD600 = 0.5) was incubated with fluorescent probe propidium iodide (PI, Cat No. C1008S, Beyotime, Shanghai, China, final concentration 10 µM) for 30 min. Then, β,β-Dim (0–32 µg/mL) was added to the PI-labeled suspension in a black 96-well plate and incubated for 1 h. Fluorescence intensity was measured at an excitation/emission wavelength of 535/615 nm. The cytoplasmic membrane permeability of *E. coli* BW25113, *E. coli* BW25113-*mcr-1*, and *E. coli* BW25113-*pmrB*R93P induced by β,β-Dim was assessed by preparing spheroplasts.

### 4.13. Effect of Membrane Components on Antibacterial Activity

The effects of membrane components on the antibacterial activity of β,β-Dim were evaluated using the checkerboard method by exogenous addition of phosphatidylethanolamine (PE), phosphatidylglycerol (PG), and cardiolipin (CL). The same procedure was followed with pre-addition of the respective components to CAMHB to explore their impact on the β,β-Dim–colistin combination in *E. coli*.

An isothermal titration calorimetry (ITC) assay was used to assess the affinity between β,β-Dim and PE, PG, and CL using NANO ITC (TA, New Castle, DE, USA). β,β-Dim (0.01 mM) was loaded into the sample cell and titrated with 20 injections of 2 μL membrane components (0.2 mM) at 120 s intervals. The assay was performed at 25 °C with a stirring rate of 350 rpm. Data were analyzed using the independent binding model to calculate the dissociation constant (*K*_D_), stoichiometric ratio (n), enthalpy change (Δ*H*), and entropy change (Δ*S*).

### 4.14. ATP Determination

Intracellular ATP levels were determined using an ATP Assay Kit (Cat No. S0027, Beyotime, Shanghai, China). Bacterial suspensions (OD600 = 0.5) were centrifuged and resuspended in PBS, β,β-Dim, colistin, or their combination and incubated for 1 h. Following treatment, the mixtures were lysed and centrifuged. Then, supernatants were mixed with the ATP detection working solution, and ATP levels were quantified by measuring relative luminescence units (RLUs).

### 4.15. Murine Abdominal Infection Model

Mice were randomly assigned to five groups (n = 10) and infected intraperitoneally with *K. pneumoniae* 667 (8 × 10^6^ CFUs). One hour post-infection, treatments were administered intraperitoneally: PBS, β,β-Dim (2 mg/kg), colistin (0.2 mg/kg), or β,β-Dim combined with colistin (2 + 0.2 mg/kg). Survival was continuously monitored. Upon death, the heart, liver, spleen, lungs, and kidney were immediately collected. At five days post-infection, surviving mice were euthanized, and the same organs were harvested. Half of each organ was weighed, homogenized, diluted, and plated on LA plates for bacterial colony enumeration. The remaining tissue samples were used for histopathological analysis.

### 4.16. Statistical Analysis

Statistical analysis was performed using GraphPad Prism 9.0 software and calculated using one-way ANOVA across multiple groups. All data were presented as mean ± SD. Statistical significance was considered as * *p* < 0.05, ** *p* < 0.01, *** *p* < 0.001, **** *p* < 0.0001.

## 5. Conclusions

In summary, this study demonstrates that β,β-Dim, in combination with colistin, effectively targets a range of colistin-resistant Gram-negative bacteria, including *K. pneumoniae*, *E. coli*, *Salmonella*, and *P. aeruginosa*, reducing colistin MICs by 4- to 1024-fold (FICI < 0.281). β,β-Dim synergistically enhances colistin activity by perturbing cytoplasmic membrane biofunction via a phospholipid interaction, further leading to membrane permeability disruption and cellular energy depletion. These changes impair LPS transport and efflux pump activity, resulting in LPS accumulation in the cytoplasmic membrane, increased intracellular colistin retention, elevated ROS production, and ultimately bacterial cell death. In vivo, the combination of β,β-Dim (2 mg/kg) and colistin (0.2 mg/kg) improved survival in a murine infection model from 20% with colistin monotherapy to 80% with combination therapy, significantly enhancing colistin efficacy in vivo. These findings identify β,β-Dim as a potent colistin adjuvant and highlight its promise as a therapeutic strategy against XDR Gram-negative bacterial infections.

## Data Availability

Data are contained within this article and Supplementary Materials.

## Supplementary Materials

The following supporting information can be downloaded at https://www.mdpi.com/article/10.3390/antibiotics15010003/s1.
