# Supplementary material for: β,β-Dimethylacrylalkannin Restores Colistin Efficacy Against mcr- and TCS-Mediated Resistant Gram-Negative Bacteria via Membrane Disturbance
Source: Antibiotics (Basel). 2025 Dec 19;15(1):3. doi: 10.3390/antibiotics15010003 (PMC12837263; doi:10.3390/antibiotics15010003)
Supplement: Supplementary file 1 [file antibiotics-15-00003-s001.zip › antibiotics-4014268-supplementary.pdf]

Supplementary materials

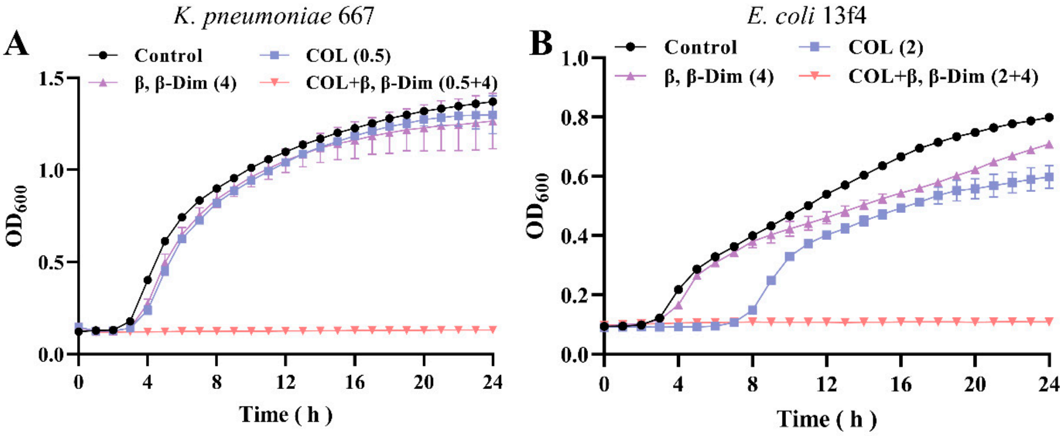

Fig. S1. Growth curves of *K. pneumoniae* 667 (C) and *E. coli* 13f4 (D) treated with the combination of β,β-Dim and colistin. COL: colistin. Data represent three biological replicates.

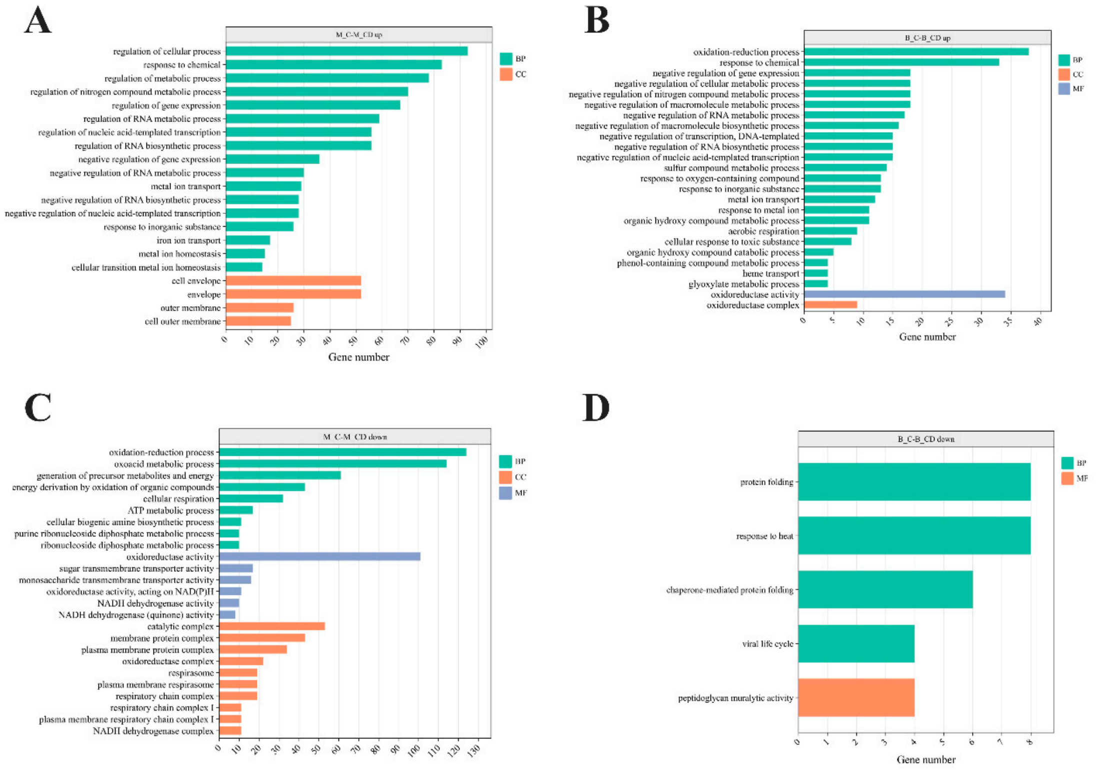

Fig. S2. GO enrichment analysis of DEGs. (A-B) GO enrichment of upregulated DEGs in *E. coli* BW25113-*mcr-1* and *E. coli* BW25113-*pmrB*R93P following treatment with β,β-Dim and colistin for 1 h. (C-D) GO enrichment of downregulated DEGs in the same strains under identical treatment conditions. M_C and M_CD: colistin group and combination group in *E. coli* BW25113-*mcr-1*. B_C and B_CD: colistin group and combination group in *E. coli* BW25113-*pmrB*R93P.

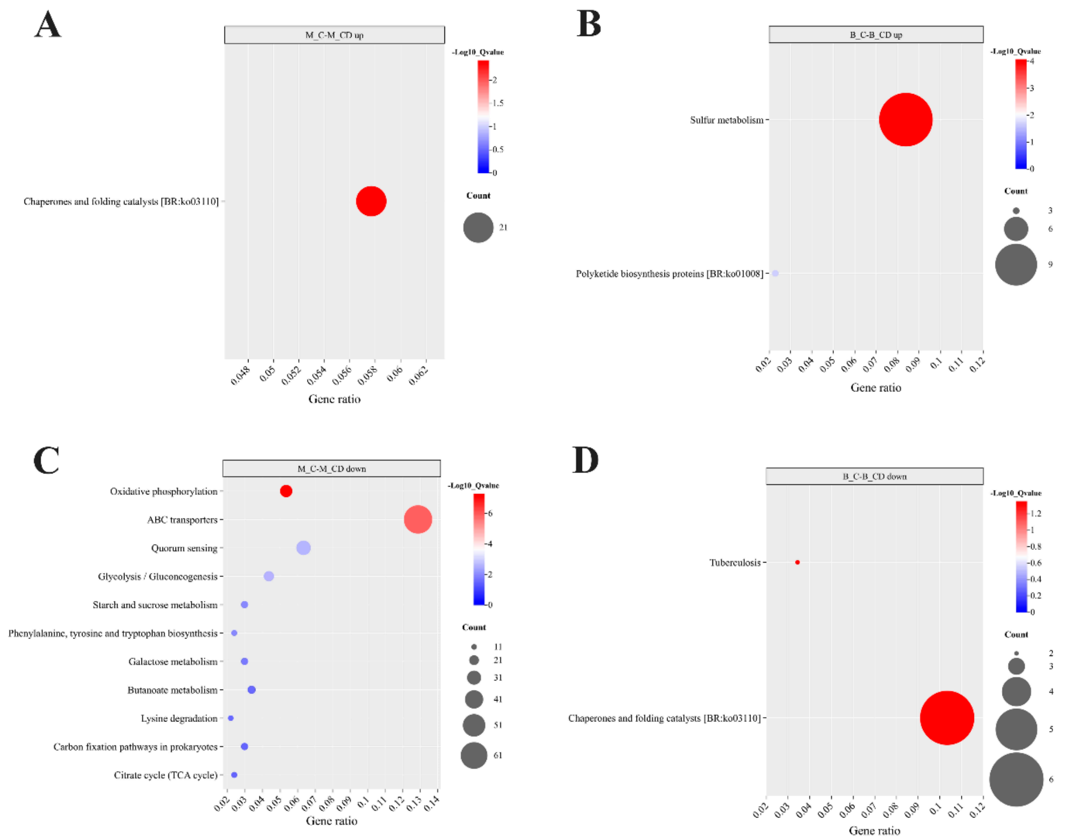

Fig. S3. KEGG enrichment analysis of DEGs. (A-B) KEGG enrichment of upregulated DEGs in *E. coli* BW25113-*mcr-1* and *E. coli* BW25113-*pmrB*R93P following treatment with β,β-Dim and colistin for 1 h. (C-D) KEGG enrichment of downregulated DEGs in the same strains under identical conditions. M_C and M_CD: colistin group and combination group in *E. coli* BW25113-*mcr-1*. B_C and B_CD: colistin group and combination group in *E. coli* BW25113-*pmrB*R93P.

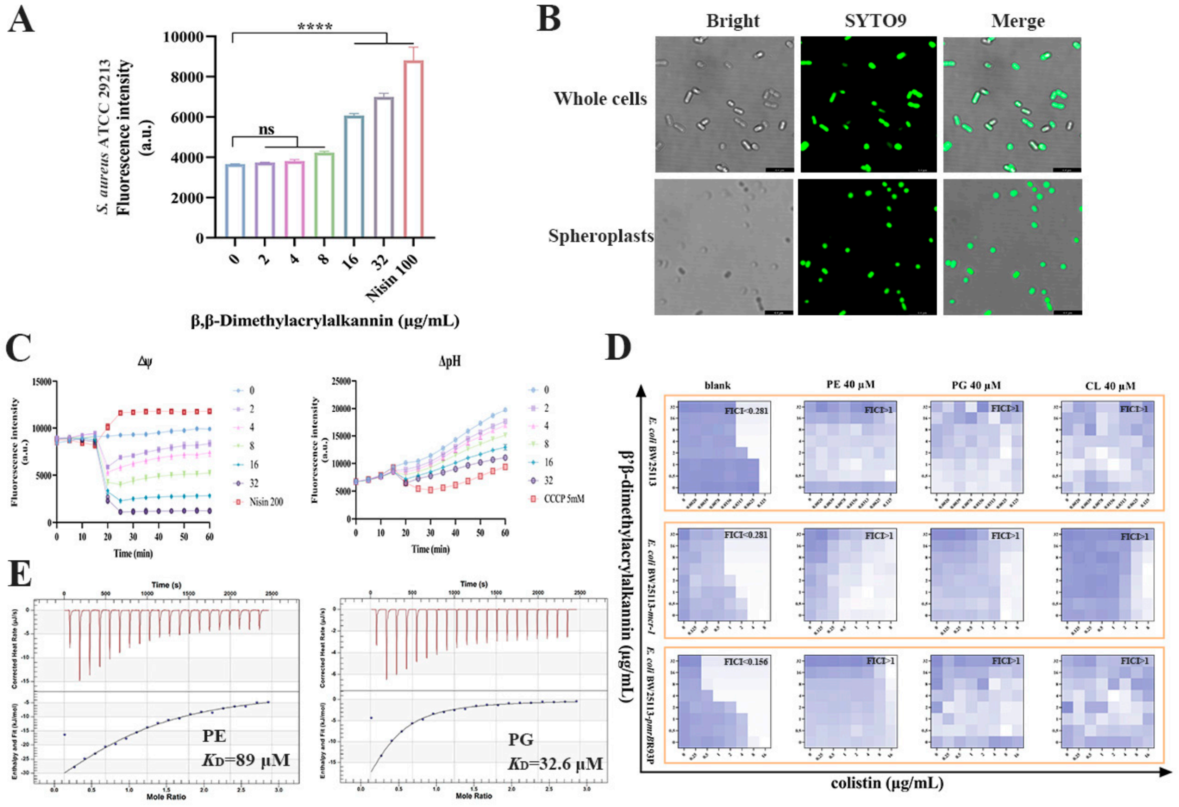

Fig. S4. Interaction of β,β-Dim with bacterial membrane components. (A) Membrane permeability in *S. aureus* ATCC 29213 after β,β-Dim treatment, nisin served as a positive control. (B) Representative CLSM images of *E. coli* whole cells and spheroplasts. Scale bars: 6.4 μm. (C) Membrane potential (Δψ) and proton gradient (ΔpH) in *S. aureus* ATCC 29213 after β,β-Dim treatment. (D) Influence of exogenous PE, PG, and CL on the synergistic activity of β,β-Dim and colistin in *E. coli*. (E) Binding affinity of β,β-Dim for PE and PG measured by ITC assays. Data represent three biological replicates. ns, no significant difference. **P* < 0.05, ***P* < 0.01, ****P* < 0.001, *****P* < 0.0001.

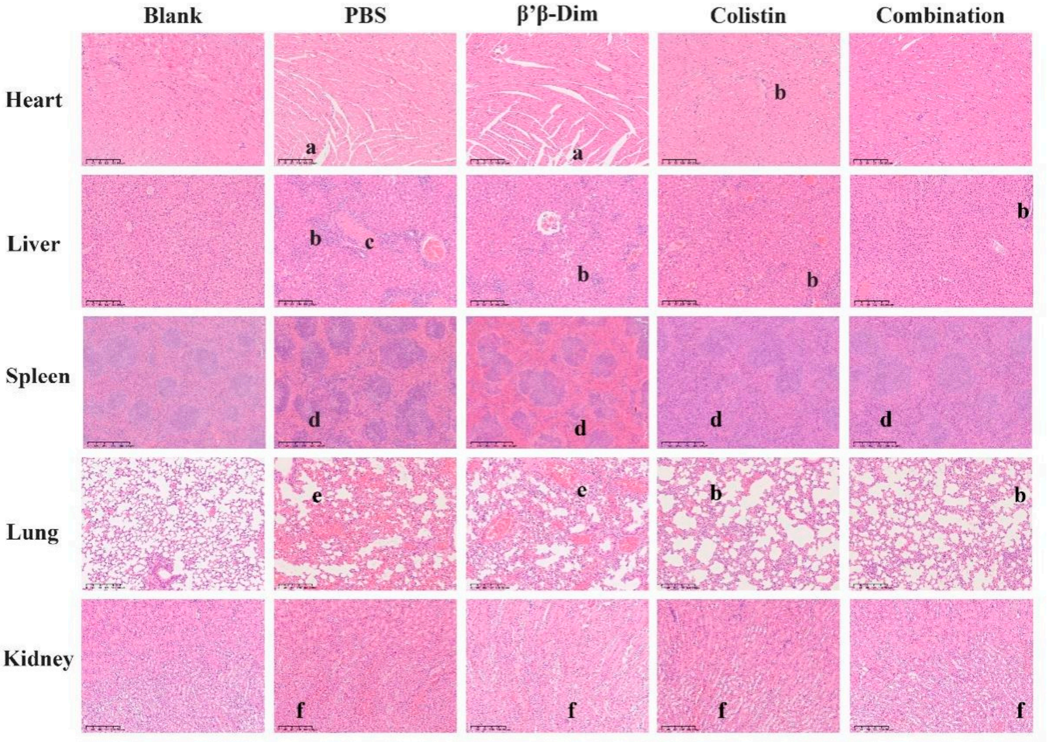

Fig. S5. Representative histopathological images of mouse tissues following treatment. a: tissue edema and interstitial widening. b: inflammatory cell infiltration. c: necrosis. d: white pulp atrophy or expansion. e: alveolar cavity congestion. f: interstitial congestion. Magnifications: heart, liver, lung, and kidney at 10×; spleen at 4×.

Table S1 The RT-qPCR primer sequences

Genes		Sequences（5'-3'）
16S rRNA	Forward	TGTAGCGGTGAAATGCGTAGA
	Reverse	CACCTGAGCGTCAGTCTTCGT
lptA	Forward	ACGCTTCCCAGATGCACTAC
	Reverse	GCTTGCCTTTGTCGCTGAAA
lptB	Forward	CGGTATCGGCTATCTGCCAC
	Reverse	CGGTCTTCACGTTGTTCAGC
lptC	Forward	AAAGTGGTCGTTACCCGTCC
	Reverse	TAGTGCATCTGGGAAGCGTG
lptD	Forward	CTGCTTGGTTGAAACGGTGG
	Reverse	ATGGATCAGGTGTGGCGTTT
lptE	Forward	CGTTCCATCCTTGCGTTTGG
	Reverse	ACGGTCGCATTAACCGTCAT
lptF	Forward	AACTGGCGTATCACGTTGGT
	Reverse	TCAGGGAGGTCTGGATCAGG
lptG	Forward	GATCTTCTTCCCGATGGCGG
	Reverse	GCGGTTTTCATCACCGACAG
marR	Forward	CGAGTATCTGTCTCCGCTGG
	Reverse	CCAGGTCGACCGACAATACC

Table S2 The MICs and FIC indexes of all the strains used in this study

Strains	Species	Source	MIC[a] [Combination]	MIC[b] [combination]	FIC index [Potentation]
13883	*K. pneumoniae*	ATCC	>128[2]	0.5[0.125]	<0.265[4]
20(*mcr-1*)	*K. pneumoniae*	engineering	>128[4]	64[1]	<0.046[64]
197(*mcr-1*、*bla*$_{NDM}$)	*K. pneumoniae*	clinical	>128[8]	4[0.5]	<0.187[8]
492(*mcr-1*、*bla*$_{NDM}$)	*K. pneumoniae*	animal	>128[2]	4[0.125]	<0.046[32]
57(*mcr-9*、*bla*$_{NDM}$)	*K. pneumoniae*	animal	>128[8]	2[0.0625]	<0.093[32]
109(*mcr-8*)	*K. pneumoniae*	clinical	>128[4]	16[0.25]	<0.046[64]
126(*mcr-8*)	*K. pneumoniae*	clinical	>128[8]	>512[0.5]	<0.063[1024]
29(*mcr-8*、*pmrB*、*bla*$_{NDM}$)	*K. pneumoniae*	clinical	>128[4]	64[0.25]	<0.035[256]
251(*mgrB*、*phoP*、*pmrA*、*bla*$_{KPC}$)	*K. pneumoniae*	clinical	>128[4]	512[0.5]	<0.032[1024]
650(*mgrB*、*phoP*、*pmrA*、*bla*$_{KPC}$)	*K. pneumoniae*	clinical	>128[8]	32[2]	<0.125[16]
667(*phoP*、*pmrA*、*bla*$_{KPC}$)	*K. pneumoniae*	clinical	>128[2]	64[0.5]	<0.023[128]
25922	*E. coli*	ATCC	>128[2]	0.125[0.0313]	<0.266[4]
ZJ71(*mcr-1*)	*E. coli*	clinical	>128[4]	4[0.5]	<0.156[8]
13f4(*mcr-1*)	*E. coli*	animal	>128[4]	4[1]	<0.281[4]
BW25113-184	*E. coli*	engineering	>128[2]	0.125[0.0313]	<0.266[4]
BW25113-184-*mcr-1*	*E. coli*	engineering	>128[4]	4[1]	<0.281[4]
BW25113-*pmrA*D82N(*pmrA*)	*E. coli*	engineering	>128[8]	16[0.5]	<0.093[32]
BW25113-*pmrB*R93P(*pmrB*)	*E. coli*	engineering	>128[8]	8[0.25]	<0.093[32]
pHSG299-*mcr-3*	*E. coli*	engineering	>128[8]	2[0.125]	<0.125[16]
14028	*S.* Typhimurium	ATCC	>128[0.5]	1[0.5]	<0.503[2]
9891(*mcr-1*)	*S.* Typhimurium	clinical	>128[8]	16[2]	<0.187[8]
PAO1	*P. aeruginosa*	ATCC	>128[2]	1[0.5]	<0.515[2]
9(*mcr-1*)	*P. aeruginosa*	animal	>128[16]	4[0.5]	<0.25[8]
19606	*A. baumannii*	ATCC	>128[4]	0.5[0.0078]	<0.046[64]

a represented the MIC of β,β-Dim for all strains, and [] represented the MIC under combination treatment. b represented the MIC of colistin for all strains, and [] represented the MIC in combination. Unit: μg/mL.

Table S3 Key differently expressed genes

Genes	Log$_2$ FoldChange	
	M_C vs M_CD	B_C vs B_CD
lptA	-1.08921	0.024207
lptB	-1.54592	0.158258
lptC	-0.63955	0.157509
lptD	-1.40942	-2.79367
lptE	-0.31206	-0.15184
lptF	0.726536	-0.38563
lptG	-0.61661	0.016158
mprA	4.924054	1.246538
marR	5.720244	2.851471
marB	3.009581	1.218489
emrA	1.102647	0.660975
emrB	0.390013	-0.45066
acrA	0.64064	0.129782
acrB	1.346519	0.488864
acrZ	2.954411	0.60667
tolC	-1.39599	0.292493

M_C and M_CD: colistin group and combination group in *E. coli* BW25113-*mcr-1*. B_C and B_CD: colistin group and combination group in *E. coli* BW25113-*pmrB*R93P.
